# Reference genome assembly for Australian *Ascochyta lentis* isolate Al4

**DOI:** 10.1093/g3journal/jkab006

**Published:** 2021-01-23

**Authors:** Robert C Lee, Lina Farfan-Caceres, Johannes W Debler, Angela H Williams, Robert A Syme, Bernadette M Henares

**Affiliations:** Centre for Crop and Disease Management, School of Molecular and Life Sciences, Curtin University, Bentley, WA 6102, Australia; Centre for Crop and Disease Management, School of Molecular and Life Sciences, Curtin University, Bentley, WA 6102, Australia; Centre for Crop and Disease Management, School of Molecular and Life Sciences, Curtin University, Bentley, WA 6102, Australia; Department of Environment and Agriculture, Curtin University, Bentley, WA 6102, Australia; Centre for Crop and Disease Management, School of Molecular and Life Sciences, Curtin University, Bentley, WA 6102, Australia; Centre for Crop and Disease Management, School of Molecular and Life Sciences, Curtin University, Bentley, WA 6102, Australia

**Keywords:** PacBio, *Pleosporales*, *Dothideomycete*, plant pathogen, lentil

## Abstract

*Ascochyta lentis* causes ascochyta blight in lentil (*Lens culinaris* Medik.) and yield loss can be as high as 50%. With careful agronomic management practices, fungicide use, and advances in breeding resistant lentil varieties, disease severity and impact to farmers have been largely controlled. However, evidence from major lentil producing countries, Canada and Australia, suggests that *A. lentis* isolates can change their virulence profile and level of aggressiveness over time and under different selection pressures. In this paper, we describe the first genome assembly for *A. lentis* for the Australian isolate Al4, through the integration of data from Illumina and PacBio SMRT sequencing. The Al4 reference genome assembly is almost 42 Mb in size and encodes 11,638 predicted genes. The Al4 genome comprises 21 full-length and gapless chromosomal contigs and two partial chromosome contigs each with one telomere. We predicted 31 secondary metabolite clusters, and 38 putative protein effectors, many of which were classified as having an unknown function. Comparison of *A. lentis* genome features with the recently published reference assembly for closely related *A. rabiei* show that genome synteny between these species is highly conserved. However, there are several translocations and inversions of genome sequence. The location of secondary metabolite clusters near transposable element and repeat-rich genomic regions was common for *A. lentis* as has been reported for other fungal plant pathogens.

## Introduction

Ascochyta blight disease of lentil (*Lens culinaris* Medik. subsp. *culinaris*) is caused by the filamentous fungal pathogen species, *Ascochyta lentis* Vassiljevsky (teleomorph: *Didymella lentis*, syn. *Ascochyta fabae* f. sp. *lentis*) (Kaiser *et al.* 1997). Symptoms of the disease in lentil include the development of necrotic lesions on leaves, stems, and pods, with consequences of reduced photosynthesis and transpiration efficiency, and seed yield and quality (Gossen and Morrall 1983). The genus *Ascochyta* includes necrotrophic pathogenic fungi that infect several of the cultivated grain legumes and each of these is specific for a particular host (Hernandez-Bello *et al.* 2006; Peever *et al.* 2007). *Ascochyta lentis* has specificity for lentil and has been shown not to cause disease symptoms on other non-host legumes such as chickpea (*Cicer arietinum* L) (Kaiser *et al.* 1997). *Ascochyta lentis* is classified within the *Dothideomycete* class, *Pleosporales* order of ascomycetes, which includes several species that cause major crop diseases of economic importance, including *Parastagonospora nodorum* and *Pyrenophora tritici-repentis* that cause foliar necrotic diseases of wheat, and *Leptosphaeria maculans*, the causal organism of canola blackleg disease (Hane *et al.* 2007; de Gruyter *et al.* 2009). Global production of lentil in 2016 was 6.3 MT and Canada and Australia, as major exporters of lentil, produced 3.2 MT (50% of global production) and 0.18 MT (3% of global production), respectively (FAOSTAT 2017). More recently, Australian production has climbed to 0.23 MT and the crop is popular with farmers in South Australia and Victoria. The cost of ascochyta blight to Australian lentil production is estimated to account for approximately 14% of crop value (Murray and Brennan 2012).

The life cycle of *A. lentis* is initiated as a seed-borne infection, or from pycnidiospores remaining in the soil or on residues from the previous lentil crop (Gossen and Morrall 1986; Tivoli and Banniza 2007). The sexual stage has been observed in Australia and may play a role in the infection cycle and long-distance dispersal of *A. lentis* (Galloway *et al.* 2004). However, the main mechanism of propagation of the disease is in repeated pycnidiospore cycles and dispersal over short distances by rain splash and wind (Tivoli and Banniza 2007). Two compatible mating types are present in *A. lentis* populations in Australia and sexual recombination leads to increased genetic diversity and rapid adaptation to newly introduced resistant lentil varieties (Ford *et al.* 2000). Plant breeding efforts in Australia have been highly successful in improving disease resistance, however, varieties such as Nipper and Northfield have become susceptible to the disease due to the increased frequency of more aggressive isolates in pathogen populations in regions where these varieties have been grown intensively (Davidson *et al.* 2016; Rodda *et al.* 2017).

Molecular mechanisms used by plant pathogenic fungi to establish and maintain host species and cultivar specificity primarily comprise plant-interacting secondary metabolite toxins (Howlett 2006; Muria-Gonzalez *et al.* 2015) and small-secreted protein effectors (de Wit *et al.* 2009; Franceschetti *et al.* 2017). For example, in the *Pleosporales* genus *Cochliobolus*, three classical small metabolite toxins have been key determinants of pathogenicity for cultivar-specific maize and oat diseases, namely T-toxin in the corn blight-causing *C. heterostrophus*, HC-toxin in *C. carbonum*, and victorin in the oat pathogen, *C. victorae* (Panaccione 1993; Condon *et al.* 2013). Also from the *Pleosporales*, *P. tritici-repentis* (Ciuffetti *et al.* 1997) and *P. nodorum* (Friesen *et al.* 2006) both carry homologous genes that encode the small, secreted protein toxin ToxA, an effector that interacts with a receptor in the host plant wheat, to confer susceptibility to the pathogen (Faris *et al.* 2010). Effectors interact with host receptors and trigger hypersensitive responses that lead to cell death, which favors necrotrophic pathogens by providing dead plant tissue from which nutrition is derived during necrotrophic growth. In biotrophs, host recognition of pathogen effectors induces effector-triggered immunity through programmed cell death of discrete cells, thus depriving the pathogen of living tissue to parasitize. In *A. lentis*, several small metabolite plant toxins have been described, including lentiquinones (Masi *et al.* 2018), and lentisone (Andolfi *et al.* 2013). The secondary metabolite, ascochytine (Kim *et al.* 2019) is produced in the *Ascochyta* species *Ascochyta pisi* and *Ascochyta fabae* and although the biosynthetic gene cluster remains largely intact in *A. lentis*, premature stop codons in key genes of the cluster preclude the production of the phytotoxic compound in *A. lentis*. Secondary metabolites from across the *Ascochyta* and closely related *Phoma* genera have been studied in detail and evidence suggests that divergence among these species is linked to the taxonomy of divergent secondary metabolite biosynthetic pathways and the resulting metabolites produced (Kim *et al.* 2016; Kim and Chen 2019). Secondary metabolite toxins may contribute to the pathogenicity of fungal phytopathogens such as *A. lentis*, however, none has been found to determine host species or cultivar specificity. The identification of protein effectors is an important aim for pathology research of ascochyta blight in lentil.

Here, we present the first genome assembly for *A. lentis* from a combination of Illumina and high-coverage PacBio SMRT sequencing data. We use the *A. lentis* genome sequence to guide the identification of effector candidate genes that may contribute to the essential characteristic of narrow host range plant pathogenic fungi. In addition, we compare *A. lentis* and *A. rabiei* genomes to look for similarities in genome architecture and homologous putative pathogenicity genes that may contribute to differences in host specificity.

## Materials and methods

### Fungal culture and DNA extraction

*Ascochyta lentis* isolate Al4 was collected by M. Nasir (Victorian Institute of Dryland Agriculture, Agriculture Victoria) at Horsham in 1998 (Nasir and Bretag 1998). Al4 is maintained at the Centre for Crop and Disease Management, Curtin University, Australia and is available by request to the corresponding authors. DNA was prepared from fungal cultures grown in 100 mL potato dextrose liquid media (for Illumina sequencing) or 80 mL yeast extract glucose (YEG) liquid media (for PacBio sequencing). Freeze-dried fungal material was ground under liquid nitrogen and DNA was extracted using the CTAB method for Illumina sequencing or using a DNA maxi-prep method as described by Xin and Chen (Xin and Chen 2012) for PacBio sequencing. RNA was digested by addition of DNase-free RNase (Sigma, St Louis, MO, USA) and DNA was resuspended in TE buffer (10 mM Tris-HCl 1 mM EDTA, pH 8). Total fungal DNA was assessed for concentration and quality using a NanoDrop Spectrophotometer (Thermo-Fisher Scientific, Waltham, MA, USA) and by agarose gel electrophoresis. Maxi-prep DNA for PacBio sequencing was prepared in 2 mL Tris-HCl, pH 8.0 and further purified using Ampure XP beads (Agencourt, Beckman-Coulter, USA) according to manufacturer’s instructions. Maxi-prep PacBio DNA was assessed for concentration and quality using Qubit and Nanodrop equipment (Thermo Fisher Scientific) and 1% agarose gel electrophoresis.

### Genome sequencing

Illumina DNA sequencing was carried out by Eurofins Genomics (Ebersburg, Germany) on the Illumina HiSeq platform (Illumina Inc. San Diego, CA, USA). Illumina 3 kb long jumping distance libraries were prepared for *A. lentis* Al4 DNA, from which 100 bp reads were sequenced. Single-Molecule, Real-Time (SMRT) PacBio sequencing was performed by Genome Quebec (McGill University, Montreal, Canada) on libraries prepared with 17 kb size-selected fragments from sheared genomic DNA. Libraries were prepared using P6-C4 chemistry and sequenced on six SMRT cells on a PacBio RSII instrument (Pacific Biosciences, Menlo Park, CA, USA).

### Reference genome assembly

The *A. lentis* Al4 genome assembly was produced from PacBio SMRT reads using the CANU assembler (v 1.2) (Berlin *et al.* 2015), and corrected and refined using Illumina short-read data with PILON v 1.2.1 (Walker *et al.* 2014). PacBio reads were trimmed and filtered in CANU using an estimated error rate of 0.03 and Illumina reads were trimmed using Trimmomatic 0.38 (Bolger *et al.* 2014). The mitochondrial genome was assembled as a single contig and was identified based on homology with mitochondrial DNA from other fungal species. Telomeres were annotated by manual observation of tandem TTAGGG repeat sequences at the ends of contigs in the final assembly. Sequencing statistics of the corrected PacBio assembly for *A. lentis* Al4 were generated using QUAST v 4.6.2 (Gurevich *et al.* 2013). Read mapping to genome assemblies was performed using Minimap2 (Li 2018) and the IGV genomics viewer (v 2.8.2) (Robinson *et al.* 2011; Thorvaldsdóttir *et al.* 2013). A subsequent analysis of assembly quality with the inclusion of varying levels of data was conducted by taking randomly selected reads representing increasing proportions of the raw reads data and producing genome assemblies using CANU (v 1.9). Telomere detection was achieved by text-based search of contig sequences for terminal telomere sequence motifs and contig length assessment was performed using QUAST. Each successive assembly with increasing levels of data input was seeded with reads from earlier assemblies and four replicate assemblies were produced at each data input level.

### Genome annotation and analysis

Prediction of gene models for the *A. lentis* Al4 genome assembly was undertaken using AUGUSTUS v 3.3 (Stanke *et al.* 2004; König *et al.* 2016) in non-comparative mode without cDNA or EST hints in the absence of specific transcriptome data for *A. lentis*. Despite not having accurate, species-specific training data, AUGUSTUS training sets include several ascomycete species that would provide a reasonable, best estimate of protein sequence predictions for *A. lentis* Al4. Evaluation of genome assembly and annotation completeness was performed using BUSCO v 4.0.5 (Simão *et al.* 2015; Seppey *et al.* 2019) using a protein fasta file from the AUGUSTUS list of annotated proteins. Benchmarking of the *A. lentis* protein set used the Ascomycota_odb10 single-copy orthologs file from the BUSCO website downloaded in September 2020 (https://busco.ezlab.org/). OcculterCut v 1.1 (Testa *et al.* 2016) was used for assessment of percent GC content across the genome and for calculating the moving average percent GC content along the assembled Al4 contigs. Descriptive statistics and histograms were generated in Excel and a Kolmogorov–Smirnov (KS) test for comparing size distributions of Al4 AT-rich and GC-balanced regions with *A. rabiei* ArME14, was performed in R. Distances of predicted carbohydrate active enzymes (CAZyme), putative effector (PE) and secondary metabolite cluster (SMC) genes to AT-rich DNA regions were calculated by including a genome feature file for these genes in OcculterCut. Analysis of variance (ANOVA) of the mean distances and a KS test for comparing the distribution of distances were performed in R.

Transposable elements (TE) and repetitive regions of the genome assembly were identified using the PiRATE Galaxy server (Berthelier *et al.* 2018) as described in the recent characterization and analysis of the *A. rabiei* reference genome (Shah *et al.* 2020). Programs for TE detection: RepeatMasker (Smit *et al.* 1996), TE-HMMER (Berthelier *et al.* 2018), MITE-Hunter (Han and Wessler 2010), SINE-Finder (Wenke *et al.* 2011), Helsearch (Yang and Bennetzen 2009), LTRharvest (Ellinghaus *et al.* 2008), TEdenovo (Flutre *et al.* 2011) and RepeatScout (Price *et al.* 2005); CD-HIT-EST (Li and Godzik 2006) for reducing redundancy; and classification using PASTEC (Hoede *et al.* 2014) to match against PiRATE Galaxy server databanks; were implemented in a stepwise process to produce a genome feature file of TE and repeat regions. For the calculation of total repetitive and TE regions, manual editing of some predicted sequences was required where overlapping elements were not identified correctly by the CD-HIT-EST redundancy reduction step. In such cases, multiple overlapping TE sequences were manually resolved to a single TE region to prevent overestimation of total TE and repetitive DNA in the cumulative base count for such types of sequences. In the supplementary genome feature file and Circos representation, all predicted TE and repetitive DNA features were included, notwithstanding a proportion of these having overlaps with neighboring TE or repeat features.

We used NUCMER v 3.1 (Kurtz *et al.* 2004) with the maxmatch setting to compare and align the *A. lentis* Al4 genome assembly to the reference assembly for the closely related species from the *Didymellaceae* family, *A. rabiei* (Shah *et al.* 2020). CAZymes were predicted from the set of putative *A. lentis* proteins using the dbCAN webserver (Zhang *et al.* 2018) and SMCs were predicted using the web-based antiSMASH fungal server, v 5.0 (Medema *et al.* 2011; Blin *et al.* 2019), as described for *A. rabiei* (Shah *et al.* 2020). Putative effector proteins were identified based on selection criteria including the presence of a secretion signal as determined using SignalP version 5.0 (Almagro Armenteros *et al.* 2019) and DeepSig (Savojardo *et al.* 2018), mature polypeptide molecular weight, number of cysteines, and EffectorP 2.0 score (Sperschneider *et al.* 2016, 2018). Mature polypeptide molecular weight was calculated in SignalP and DeepSig and where there was disagreement about the signal peptide processing site, the SignalP prediction was favored. Effector selection data for the complete set of protein sequences were processed using a custom Python pipeline [Johannes Debler. (2019, November 4). JWDebler/effector_selection: First working release (Version v1.0) Zenodo. http://doi.org/10.5281/zenodo.3526820], where molecular weight and EffectorP thresholds were set at 15 or 25 kDa, and 0.8, respectively. Circos (Krzywinski *et al.* 2009) was used for graphical representation of features of the *A. lentis* genome assembly, and sequence homology and synteny with the closely related *Dothideomycete* species, *A. rabiei* using the links function.

### Data availability

Illumina sequence data for *A. lentis* Al4 is deposited at Sequence Read Archive (SRA) under accession number SRX5179495. PacBio SRA data is listed under accession number SRX5172313. The GenBank assembly accession number for the final genome assembly and annotation for *A. lentis* Al4 is GCA_004011705.1. BioProject and BioSample numbers for *A. lentis* Al4 are PRJNA510691 and SAMN10613129, respectively. Supplementary material is available at figshare: https://doi.org/10.25387/g3.12637415.

## Results and discussion

### Near-complete genome assembly of *Ascochyta lentis*

**Figure 1:**
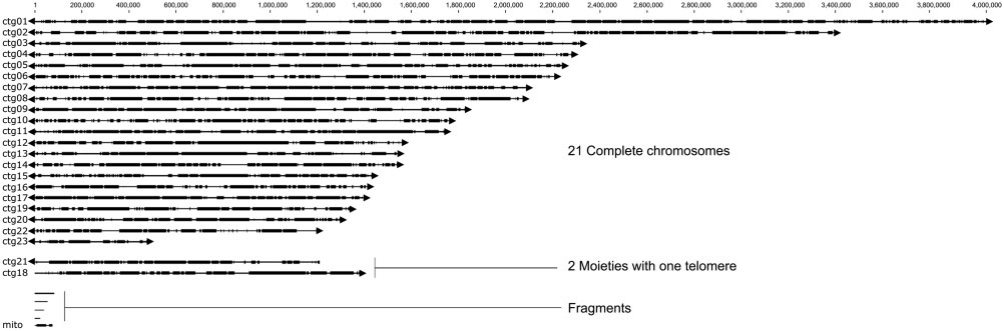
Genome contigs for the reference assembly of *Ascochyta lentis* Al4, produced from PacBio SMRT sequencing. Nuclear contigs are labeled ctg01 to ctg23 as archived in NCBI BioProject PRJNA510691 and the mitochondrial contig is labeled mito. Gene-dense regions of the genome are shown as dark-shaded blocks, and gene-sparse and interspersed repeat-rich regions are indicated by light-colored lines. Telomeres are indicated in the figure by triangles at contig ends where they were identified.

Using a combination of PacBio SMRT sequencing and correction with Illumina short reads, we have produced a near-complete genome assembly for the Australian *A. lentis* isolate, Al4. Sequencing statistics are shown in Table 1. Sequencing coverage on six SMRT cells was 288x and led to a high-quality assembly of 41,975,033 bp, consisting of 21 complete nuclear chromosomal contigs with telomere sequences at both ends (contigs 1 to 17, and contigs 19, 20, 22, 23) and two large partial chromosomal contigs with one telomere (contigs 18 and 21). Figure 1 shows *A. lentis* Al4 contig sizes in scale and four smaller assembled fragments (contigs 24-27) with sizes ranging from approx. 25 to 81 kb. These smaller fragments were highly repetitive and only represent a small fraction of the assembly (∼200 kb, 0.5%). The assembly contained a single mitochondrial genome of 73,356 bp which is similar to the mitochondrial genome size of 74,172 bp for *A. rabiei* (Shah *et al.* 2020). Telomeres were identified as TTAGGG repeat sequences at contig ends, as has been reported for filamentous fungi such as *Neurospora crassa* (Schechtman 1990), *Cladosporium fulvum* (Coleman *et al.* 1993), and *Magnaporthe oryzae* (Rehmeyer *et al.* 2006; Farman 2007). Overall GC content for *A. lentis* Al4 was 48.4%, which is similar to the GC content determined for *A. rabiei* (49.2%) (Shah *et al.* 2020). The complete assembly size of almost 42 Mb for *A. lentis* Al4 is around 1.05 Mb larger than *A. rabiei* (Shah *et al.* 2020). Although no previous studies have estimated the genome size or the number of chromosomes for *A. lentis*, Akamatsu *et al.* (2012) found *A. rabiei* isolates to contain 12 to 16 chromosomes ranging from 0.9 to 4.6 Mb in size and complete genome size ranged from 23 Mb to 34 Mb. PacBio sequencing of two closely related *Ascochyta* species, *A. rabiei* (Shah *et al.* 2020) and *A. lentis* herein, suggests that *Ascochyta* genome size is larger than was previously determined using electrophoretic karyotyping. Examples of closely related fungal species with similar chromosome number and genome size include the *Leotiomycete* fungi *Botrytis cinerea* (van Kan *et al.* 2017) and *Sclerotinia sclerotiorum* (Derbyshire *et al.* 2017), both having 16 core chromosomes with genome sizes of 43 Mb and 39 Mb, respectively. In *B. cinerea*, there are two small accessory chromosomes of 247 and 209 kb (van Kan *et al.* 2017) and these are not present in *S. sclerotiorum* (Derbyshire *et al.* 2017). In contrast, whereas the two barley net blotch-causing *P. teres formae speciales* both have 12 chromosomes, their overall genome size varies from 39–41 Mb for *P. teres* f. *maculata* to 46–50 Mb for *P. teres* f. *teres* (Syme *et al.* 2018). The larger genome size for *P. teres* f. *teres* is due to greater transposon activity and expansion of transposon-affected regions of the genome that has occurred for *P. teres* f. *maculata* (Syme *et al.* 2018). With the growing number of whole-genome sequencing projects for filamentous fungi and in particular, filamentous fungal phytopathogens, hypotheses about the correlation of genome size and the ability for species to adapt to selection pressures of their respective hosts suggest that larger genomes are more flexible and able to adapt across time (Raffaele and Kamoun 2012). In addition to other changes to genome architecture such as duplication and hybridization, fungal genomes can become larger due to expansion of TEs and repetitive DNA. These mechanisms potentially contribute to duplication of pathogenicity genes such as effectors, and provides the basis for evolution of molecular mechanisms of adaptation in the pathogen-host relationship (Raffaele and Kamoun 2012; Dong *et al.* 2015; Thynne *et al.* 2015; Syme *et al.* 2018). The number of TEs and repetitive regions within fungal genomes are by no means conclusively linked to increased gene diversity for pathogenicity genes, and other evolutionary mechanisms such as DNA mutation, genetic drift and sexual recombination are likely equally important factors in phytopathogen evolution.

**Table 1:** Summary of assembly and annotation statistics for PacBio SMRT sequencing of *Ascochyta lentis* isolate, Al4

Statistics	*Ascochyta lentis* Al4 PacBio SMRT
Assembly statistics	
Genome size (bp)	41,975,033
Sequencing reads	980,892
Total sequenced bases	12.1 Gb
Coverage	288x
Number of contigs	28
Largest contig size (bp)	4,041,206
L50	9
N50 (bp)	1,825,080
GC content	48.4 %
% TE and repeat sequence^a^	18%
Mitochondrial genome size (bp)	73,356
Complete nuclear chromosomes	21
Annotation statistics	
Number of protein coding genes	11,638
Predicted secreted proteins	1,251
Predicted effectors	38
Predicted sec. metabolite clusters	31
Predicted no. of CAZymes	491

a 7,588,490 bp.

It is possible that large *A. lentis* contigs 18 and 21 are two partial and separate chromosomes. Alternatively, they could be two ends of a single chromosome that could not be assembled due to a lack of intervening sequence data. Nevertheless, the Al4 genome comprises 22 to 23 chromosomes. PacBio sequencing at 288x coverage with Illumina correction provides a high-quality *A. lentis* Al4 genome assembly with the 23 largest contigs representing a near-complete reference genome for the species. Further accuracy of the assembly could be achieved using optical mapping in conjunction with PacBio sequencing, as has been used for the fungal phytopathogen, *Verticillium dahlia*e (Faino *et al.* 2015), and *B. cinerea* (van Kan *et al.* 2017) and this might be a future research objective. The high level of PacBio sequencing depth at 288x for *A. lentis* has allowed us to assess the appropriate level of sequencing required to produce a high quality, near-complete fungal genome assembly. We assembled the *A. lentis* genome sequence using increasing amounts of randomly selected raw sequencing reads and determined that the assembly size and the number of complete chromosomal contigs with two telomeres reached a maximum for assembly using 50 percent of the data and was not substantially improved by inclusion of higher amounts of data (Supplementary Figure S1). From this analysis we conclude that three SMRT cells and around 140x coverage would have been sufficient to produce the near-complete genome assembly that we present in the current study.

### *Ascochyta lentis* genome annotation and repeat content

**Figure 2:**
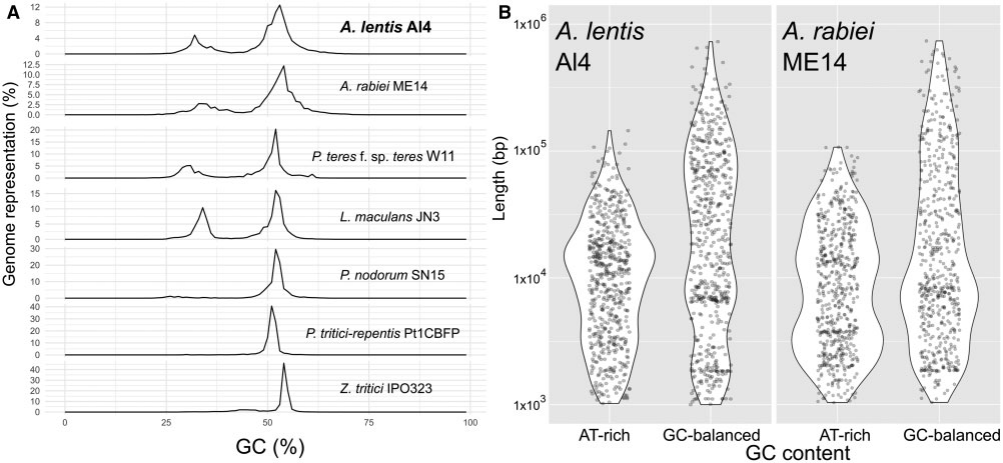
(A) GC content (%) distribution for *Dothideomycete* genome assemblies including *Ascochyta lentis* Al4. (B) Size distribution of AT-rich and GC-balanced regions for *A. lentis* Al4 and *A. rabiei* ArME14.

AT-rich DNA sequence in fungi arises largely as a result of repeat-induced point mutation (RIP) where repetitive DNA resulting from transposon insertions in the genome is targeted by the RIP mechanism that mediates cytosine to thymine base transition (van de Wouw *et al.* 2010; Rouxel *et al.* 2011; Grandaubert *et al.* 2014; Testa *et al.* 2016). Methylated cytosine residues at CpG sequences in vertebrates (Tomkova and Schuster-Böckler 2018) and yeasts (Behringer and Hall 2016) can be delaminated to thymidine residues and this is potentially an additional mechanism for mutation and increase in AT content of genomic regions in filamentous fungi. Along the course of evolution of fungal species, such nucleotide conversion leads to reduced GC content in genomic regions that often have a high frequency of TEs. OcculterCut software (Testa *et al.* 2016) determines regions of alternating gene-sparse, AT-rich DNA sequences and GC-balanced regions that contain protein coding genes. Figure 1 shows GC-balanced gene-containing regions of the Al4 genome assembly as thick, black-shaded blocks alternating with AT-rich genomic regions represented by connecting lines. Figure 2 shows the size distributions of AT-rich and GC-balanced regions in the *A. lentis* Al4 genome compared with other *Dothideomycete* genomes. Figure 2B shows that *A. lentis* is similar to the closely related species, *A. rabiei* for percent GC, the number and for the overall total base count of AT-rich and GC-balanced regions of their genomes. However, the KS test for comparing the size distributions of AT-rich and GC-balanced regions showed that there were differences between species, with a tendency toward a higher frequency of longer regions of both AT-rich and GC-balanced sequence in *A. lentis*. Further statistical analysis of the size distributions of GC-balanced and AT-rich DNA is provided in Supplementary Figure S2. Annotation of TE and repeat sequences for *A. lentis* produced a substantial number of overlapping sequences that were edited and merged manually for instances where a sequence was identified more than once. Table 2 shows the types and numbers of TE and repetitive elements for *A. lentis* Al4. The numbers and sizes of the different classes of TEs and repetitive sequences between *A. lentis* Al4 and *A. rabiei* ArME14 (Shah *et al.* 2020) were reasonably similar. Minor variations in the complement of TEs could be ascribed to differences in the annotation and classification processes performed by the PiRATE Galaxy server for the two species, or possibly to genome evolution after speciation in one or both species. For the most highly represented TE classes in Al4 and ArME14, there was some variability in the number and length of annotated elements. For example, there were similar numbers for the Class I long terminal repeats (LTR) for Al4 (694) and ArME14 (780), but the average size was higher for Al4 (7.4 kb) than for ArME14 (4.3 kb). Class I long-interspersed element (LINE) TEs were less abundant in Al4 (24 elements) than in ArME14 (177 elements) although longer in Al4 (4.2 kb) than in ArME14 (2.5 kb). For the Class II TEs, terminal inverted repeats (TIR) and Helitron TEs were in lower abundance in *A. lentis* Al4 than in *A. rabiei* ME14 although both element types were longer in *A. lentis*.

**Table 2:** Transposable element and repetitive DNA sequences from *Ascochyta lentis* Al4 determined using the PiRATE Galaxy Server

Class	Type	Number of sequences	% of total sequences	Total nucleotides	% of total nucleotides	Average size
**I**	LTR	694	52	5,113,382	67	7,368
	LARD	24	1.8	123,898	1.6	5,162
	LINE	7	0.5	29,072	0.4	4,153
	DIRS	2	0.1	20,980	0.3	10,490
	TRIM	4	0.3	8,490	0.1	2,123
	SINE	0	0.0	0	0.0	N/A
**II**	TIR	250	18.5	1,050,867	14	4,204
	Maverick	2	0.1	8,655	0.1	4,328
	Helitron	2	0.1	17,343	0.2	8,672
	MITE	6	0.4	3,736	0.05	623
	No Cat	316	23.4	1,083,162	14	3,428
	Host gene	26	1.9	118,296	1.6	4,550
	SSR	15	1.1	10,609	0.1	707
**Total**		1,348^a^	100	7,588,490	100	

a Note: 2,800 repeat and transposable element regions were annotated but after manual annotation and merging of overlapping sequences, 1,348 regions were found. All 2,800 sequences are listed in the supplementary genome feature file.

### Gene annotation and effector, secondary metabolite cluster and CAZyme prediction

Gene finding and annotation using AUGUSTUS (Stanke *et al.* 2004, 2006; König *et al.* 2016) in non-comparative mode led to the prediction of 11,638 gene models. This was close to the number of 11,257 genes that were predicted for *A. rabiei* (Shah *et al.* 2020) using AUGUSTUS gene prediction with cDNA hints from *in vitro* and *in planta*, cDNA and RNA sequence data from *A. rabiei* sequencing projects (Fondevilla *et al.* 2015). Assessment of assembly and annotation completeness using BUSCO found eight missing genes, two fragmented and one duplicated gene from the Ascomycota single-copy orthologs set of 1706 protein-coding genes (Supplementary Figure S3). Numbers of secreted proteins (1251), predicted effectors (38), secondary metabolite clusters (31) and CAZymes (491) (Table 3) were close to estimated protein numbers for *A. rabiei* (1145 secreted proteins, 39 effectors, 26 secondary metabolite clusters and 451 CAZymes) (Shah *et al.* 2020). Putative effector genes were defined as the set of *A. lentis* Al4 proteins predicted to be secreted by SignalP and DeepSig, small (<25 kDa mature MW) and have an EffectorP 2.0 score above 0.8 (Sperschneider *et al.* 2016, 2018). A set of 38 proteins fitted these criteria and their details are listed in the Supplementary data table, File S2. From the set of predicted effectors, seven protein sequences had no tBLASTn hit in the NCBI NR database, three were CAZymes and 21 proteins had no annotated function. Proteins with tBLASTn hits that have functional descriptions included two possible Cytochrome P450 proteins, two *Alternaria* major allergen AltA1 proteins, and proteins described as endosomal cargo receptor and yeast killer toxin homolog. PE25 is a homolog of the known fungal necrotrophic effector NEP, first characterized from *Fusarium oxysporum* (Bailey 1995). There is a second NEP paralog in *A. lentis* Al4, but the EffectorP 2.0 score for the second paralog fell below the 0.8 cut-off for this study, with a score of 0.61. Secondary metabolite clusters were identified in *A. lentis* Al4 and several of these were predicted by the program antiSMASH to be homologous to known phytotoxin biosynthesis genes for production of *L. maculans* sirodesmin (Gardiner *et al.* 2004), *C. carbonum* HC-toxin (Scott-Craig *et al.* 1992), *C. heterostrophus* T-toxin (Yang *et al.* 1996), ascochytine from *A. fabae* (Kim *et al.* 2019), melanin from *A. rabiei* (Akamatsu *et al.* 2010) and mellein from *P. nodorum* (Chooi *et al.* 2015). These clusters are large and complex and nucleotide sequence homology with characterized clusters in other species should be considered with caution. The aforementioned disruption of ascochytine synthesis genes in *A. lentis* is an important example of where a biosynthetic gene cluster has been modified to change the types of metabolites produced with the potential to alter pathogen host interactions (Kim *et al.* 2019).

**Table 3:** Summary of key secondary metabolite clusters, predicted effectors and CAZyme classes identified from the *Ascochyta lentis* Al4 genome assembly

Class	Number
Secondary metabolite clusters	
Total	31
T1PKS	13
T3PKS	0
NRPS	5
NRPS-like	5
NRPS/NRPS-like—T1PKS	5
Indole	1
Terpene	2
Effectors	
EffP > 0.8, MW < 25 kDa^a^^,b^	38
EffP > 0.8, MW < 15 kDa^a^	21
CAZymes	
Total	491
AA—Multicopper oxidases	88
CBM—Carbohydrate-binding module	5
CE—Carbohydrate esterase	34
GH—Glycoside hydrolase	251
GT—Glycosyl transferase	80
PL—Polysaccharide lyase	33

Detailed tables are provided in the Supplementary data.

a Mature protein MW

b Average MW = 14.46, average EffP score = 0.90

**Figure 3:**
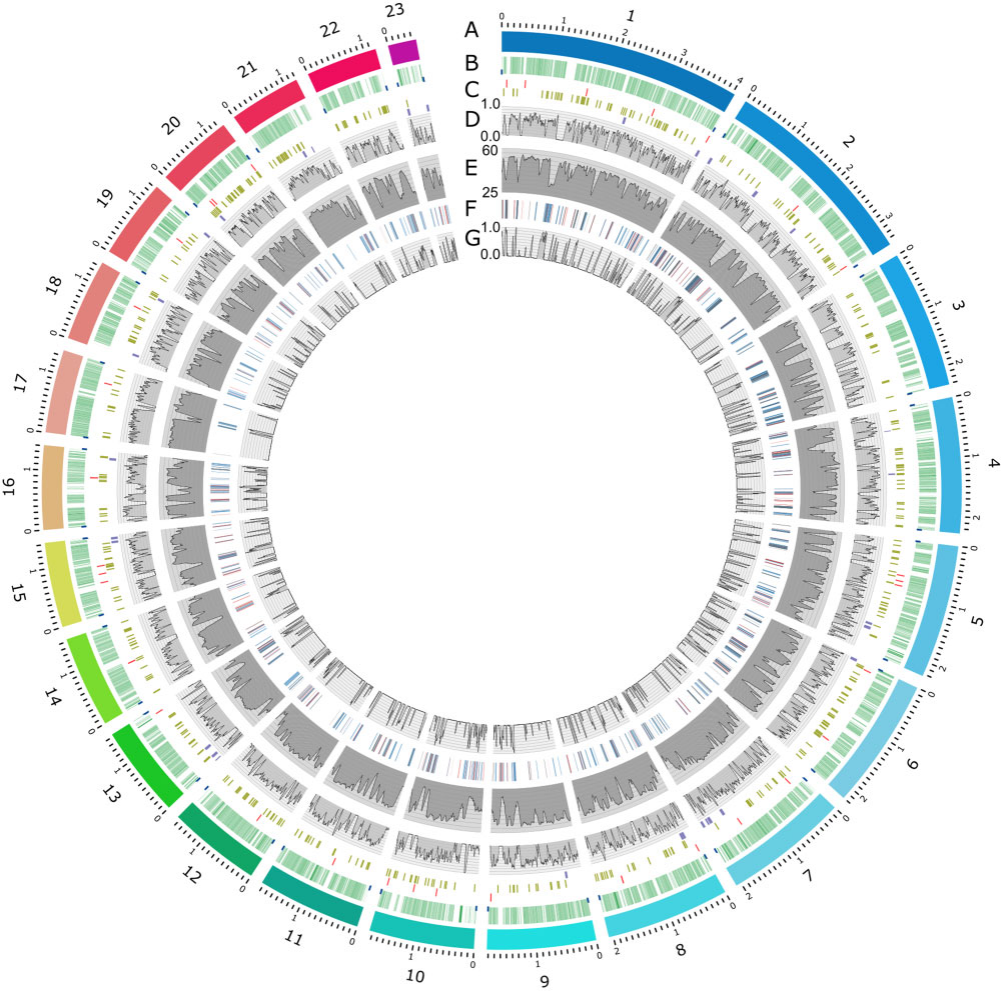
Circos plot of key features of the *Ascochyta lentis* Al4 reference genome. Tracks labeled from outside: (A) The 23 largest contigs with size scale in Mb; (B) Annotated genes (green) and telomeres (not to scale) (blue); (C) Locations (not to scale) of predicted effector genes (red), CAZyme genes (green), and predicted secondary metabolite clusters (purple and in scale); (D) Gene density (20 kb moving average window); (E) % GC content (50 kb moving average window); (F) Transposable elements and repetitive DNA sequence regions. LTR, LARD, LINE, DIRS and TRIM elements (blue), TIR, MITE, Helitron and Maverick elements (red), SSRs (orange), No category and potential host gene (gray); (G) TE and repetitive DNA density (20 kb moving average window). An SVG version of this figure (Figure_S5.svg) is included in the Supplementary material to enable closer inspection of genome features.

Figure 3 shows the arrangement of *A. lentis* chromosomes and key genome features in a Circos plot representation. The 23 largest contigs are shown as differently colored blocks (track A), and annotated genes and telomeres are indicated in track B. The locations of genes putatively associated with the plant pathogenic lifestyle of *A. lentis*, including predicted effectors, CAZymes and secondary metabolite biosynthetic gene clusters are indicated in track C. Regions of TE and repetitive DNA sequence (tracks F and G) correspond with an absence of predicted genes (tracks B and D) and low percent GC content (track E). CAZymes and predicted effector gene locations (track C) were distributed evenly across GC-equilibrated regions of the genome, and SMCs were biased toward the sub-telomeric regions at the ends of contigs, or within or adjacent to transposon and repeat-rich DNA. Examination of the proximity of SMC and effector genes to these regions found that 66% of putative effector genes and 84% of SMCs were within 50 kb of an AT-rich, sub-telomeric or intra-chromosomal region of the genome. Full data analysis for the distance from predicted effector genes and SMCs to the nearest AT-rich DNA region is presented in Supplementary Figure S4. AT-proximity of putative pathogenicity genes that comprise the predicted effector genes and likely a proportion of the SMCs was compared to CAZyme genes that we presumed to be more randomly distributed across the genome. The location of SMCs was biased toward close proximity to AT-rich DNA regions, with 18 of the 31 SMCs being located directly adjacent to AT-rich DNA and a further five being within 10 kb. Eleven putative effector genes of 38 were within 10 kb of an AT-rich region and a further 17 were within 50 kb. KS tests showed that the distribution of distances from SMCs to AT-rich DNA was significantly different to CAZyme and putative effector genes (*p* < 0.05). However, mean and distribution of distances for CAZymes and putative effectors were not significantly different from each other (KS statistic *D* = 0.14; *P* = 0.49). Proximity to transposons has been proposed as a mechanism for mobility of effector genes, by which genes can be duplicated and transferred between species for some plant pathogenic fungi (Rouxel *et al.* 2011). However, our data show that in *A. lentis*, this close proximity only stands for a minor proportion of the predicted effector genes. Repeat-induced point mutation has been reported as an additional driver of genome variation and evolution of host pathogenicity genes through RIP-dependent mutation in genomic sequence close to repetitive DNA (Fudal *et al.* 2009; Rouxel *et al.* 2011). It is unclear whether the genomic context of the proposed pathogenicity genes in *A. lentis*, particularly the putative effector genes, has had any effect on their evolutionary history or their putative role in disease.

### Comparison of the *A. lentis* and *A. rabiei* genomes shows strong syntenic relationship and evidence of chromosomal rearrangement

**Figure 4:**
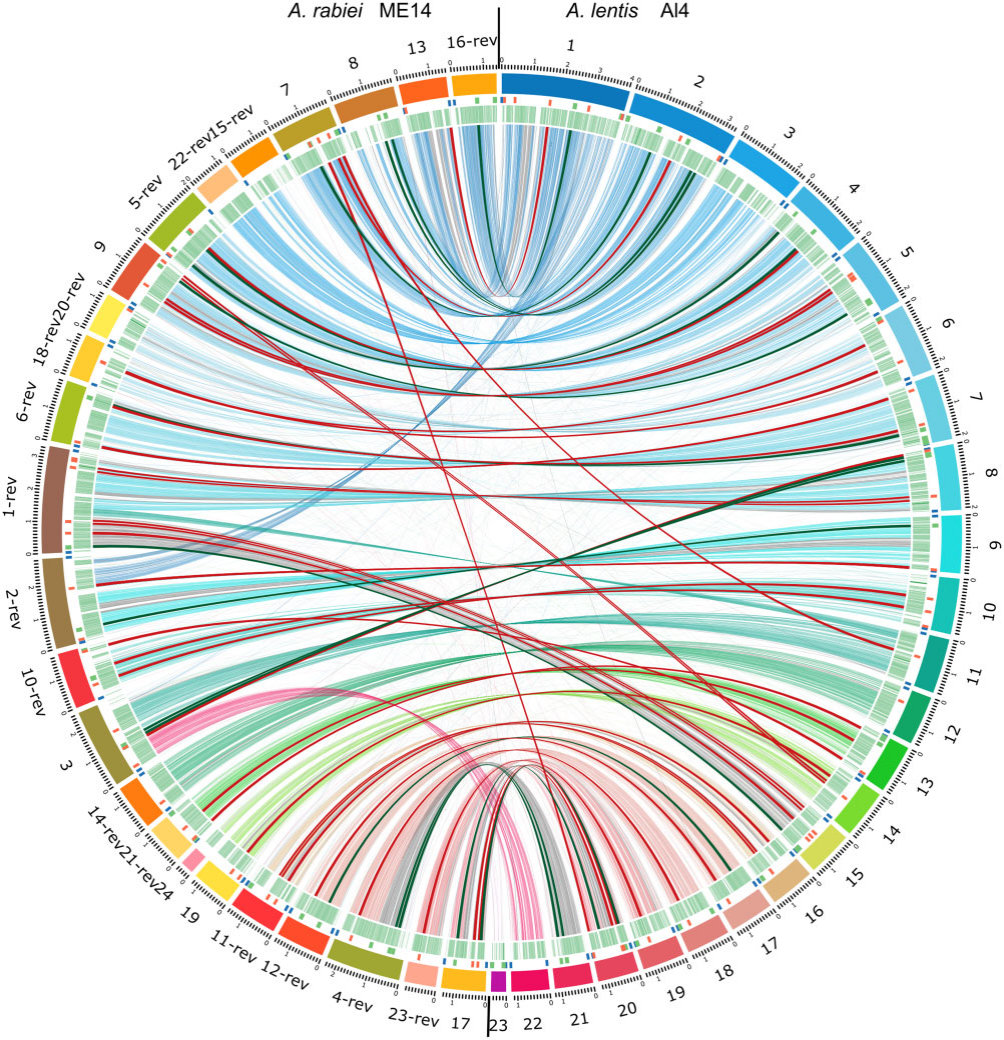
Synteny between *Ascochyta lentis* Al4 (right side) and *A. rabiei* ArME14 (left side) genome assemblies. Homologous regions were determined using NUCMER and plotted using Circos. *A. rabiei* contigs were reordered and in some cases reversed, to align with *A. lentis* assembly contigs that were arranged in order from largest to smallest. Between the outside contigs track and the links, are locations of putative effectors (red), telomeres (blue) and SMCs (green), and annotated genes (green). Locations of homologous putative effector and SMC genes in Al4 and ArME14 are indicated by red and green links (not to scale), respectively. An SVG version of this figure (Figure_S6.svg) is included in the Supplementary material to enable closer inspection of genome synteny between species.

In closely related plant pathogenic fungi, gene sequence differences and presence-absence variations for key pathogenicity genes between species will likely influence host and cultivar specificity. Figure 4 shows synteny between *A. lentis* Al4 and *A. rabiei* ArME14 genome assemblies represented as links between homologous genomic sequences in the combined Circos plot of the two genome assemblies. Unique homologous sequences between genomes were matched using the NUCMER program (Kurtz *et al.* 2004) and processed for graphical representation using the links function in Circos. Homologous sequence (>75% nucleotide identity) between the species amounted to 16.6 Mb, or 40% of the two similarly sized genomes in 9144 blocks of matched sequence, and synteny was highly conserved. *A. lentis* contigs are presented in order of decreasing size in a clockwise direction in Figure 4. *A. rabiei* ArME14 contigs as extracted from GenBank (GCA_004011705.1) were arranged in forwards or reverse-compliment orientation (notated with the suffix –rev) to present homologous contigs in the same order as for *A. lentis*, in an anticlockwise direction. Links are colored according to the color of the respective *A. lentis* contig track and links in gray indicate inversion of sequence within contigs, between *A. lentis* and *A. rabiei*. Syntenic relationships illustrated in this way suggest that partial chromosome contigs of the *A. rabiei* assembly may in fact be joined together, although in most cases the position of telomeres for ArME14 would preclude merging of ArME14 contigs in this way.

Homology exists and synteny is preserved between Ar-13/Ar-16-rev, and Al-1; Ar-7/Ar-8 with Al-2; Ar-22-rev/Ar-15-rev with Al-3; Ar-18-rev/Ar-20-rev with Al-6; and Ar-24/Ar-19 with Al-14. Nine direct contig matches exist between Ar-9 and Al-5; Ar-6-rev and Al-7; Ar-10-rev and Al-10; Ar-14-rev and Al-12; Ar-21-rev and Al-13; Ar-11-rev and Al-16; Ar-12-rev and Al-17; Ar-23-rev and Al-19; and Ar-17 and Ar-20. Ten large inversions of DNA sequence are present between homologous *A. rabiei* ArME14 contigs and *A. lentis* Al4 contigs: Al-1 (300 kb), Al-4 (420 kb), Al-5 (130 kb), Al-7 (230 kb), Al-8 (250 and 160 kb), Al-9 (310 kb), Al-14 (70 kb), Al-16 (160 kb), and Al-20 (280 kb). Major chromosomal rearrangements were identified between *A. lentis* Al4 and *A. rabiei* ArME14, with 960 kb from Al-1, translocated in the homologous *A. rabiei* contig Ar-2-rev, and 270 kb from Al-11 translocated to Ar-1-rev in *A. rabiei*. Full-length homologous chromosomes with two telomeres in *A. lentis*, Al-15, Al-21, and Al-22, were joined with other contigs in homologous *A. rabiei* chromosomes. The annotation of telomere sequences at both ends for some of the ArME14 contigs suggests that they are not conjoined despite the homology and synteny with long chromosomal contigs from *A. lentis*. The inversion and translocation of chromosomal segments in *A. rabiei* and *A. lentis* revealed by PacBio sequencing are notable features of these two members of the *Ascochyta* genus. In filamentous fungal pathogens, chromosomal rearrangement by sectional translocation or inversion is a mechanism for species evolution and adaptation to new host plant species or cultivars, both between and within pathogen species (Raffaele and Kamoun 2012; de Jonge *et al.* 2013; Grandaubert *et al.* 2014). Within the *formae speciales* of *P. teres*, *P. teres* f. *teres* has undergone major expansion of TE and repetitive regions compared with *P. teres* f. *maculata* but there were no chromosomal rearrangements or sectional inversions between the two species (Syme *et al.* 2018). Contrasting with this, within the species *V. dahliae* different isolates have undergone extensive chromosomal rearrangement and this is suggested to have influenced host specificity through deletion and insertion of transposon-associated genes at inter- and intrachromosomal breakpoints (de Jonge *et al.* 2013). Intrachromosomal inversions were common in *L. maculans* and related species *Leptosphaeria biglobosa*, as revealed by sequence alignment and comparison of the two genome assemblies (Grandaubert *et al.* 2014). Sectional translocations, as we have observed in *A. lentis* and *A. rabiei*, were not evident in the two *Leptosphaeria* species (Grandaubert *et al.* 2014). *A. lentis* undergoes both asexual and sexual reproduction (Gossen and Morrall 1986; Tivoli and Banniza 2007). Sexual reproduction provides a means of enabling rapid pathogen adaptation and possibly increased pathogenicity. It is unclear from our high-quality assembly of only one *A. lentis* isolate whether the mode of reproduction may have affected the observed chromosomal rearrangements between *A. lentis* and *A. rabiei*. These changes in genome architecture are more likely to have accumulated gradually over the extended period of species divergence.

A BLASTp search of 38 putative *A. lentis* effectors against an *A. rabiei* ArME14 effector database of 39 putative effectors with EffectorP score above 0.8, found 18 direct orthologs, with protein sequence identity from 52% to 95% (Supplementary file, File S2). In Figure 4, putative effector orthologs from *A. lentis* and *A. rabiei* effector lists are indicated by links shown in red. Six of the 18 ortholog pairs were found in non-syntenic locations between *A. lentis* and *A. rabiei*. Such translocated putative effector genes were always located near repetitive DNA sequence or contig ends. The other 12 *Ascochyta* orthologs were in conserved syntenic regions between species. For Al4 effectors with no BLASTp hit to the ArME14 effectors list, eight matched the ArME14 proteins list using BLASTp (71% to 90% protein sequence identity). These eight ArME14 orthologs were not on the ArME14 effectors list, having EffectorP scores of less than 0.8 (0.58–0.79), but were almost all secreted proteins (one exception) and of small size (mature MW 10.4–34.4 kDa). Eight Al4 putative effectors had no homologous *A. rabiei* sequence by either BLASTp to ArME14 effector and protein lists, or by tBLASTn to the ArME14 genome assembly, and these might be novel effector candidates that are unique to *A. lentis* in the *Ascochyta* genus. Some of these candidates with no orthologous gene sequence in ArME14 do have orthologs in other plant pathogenic fungi. This further emphasizes the mobility of effector genes, gene loss or gain, and protein sequence evolution that marks the role of effectors in the co-evolution of plant pathogens and their hosts. As a corollary to these features of effector genes, comparing homologous candidate effector genes among closely related and more distant species can assist the selection of genes that may determine host species preference in host-specific pathogens. Links between orthologous Al4 and ArME14 annotated proteins predicted to be an effector (EffectorP score greater than 0.8) in one species but not the other (EffectorP score below 0.8) are also shown as red links in Figure 4. Seven of these additional orthologous putative effectors were in non-syntenic locations between the two genome assemblies, on contigs Al-6, Al-8, Al-11, Al-13, Al-14 (x2), and Al-20.

In the *A. lentis* list of putative effectors there were three pairs of adjacently located paralogous genes that are proteins of unknown function. These paralogous protein pairs [PE16 and PE17 (contig Al-8); PE26 and PE27 (contig Al-14); and PE31 and PE32 (contig Al-16)] had approximately 40% amino acid identity to each other (Supplementary file, File S2), and their orthologous pairs in *A. rabiei*, were located adjacently on their respective contigs (Figure 4). Although two of these pairs were in regions of conserved synteny, one pair of neighboring paralogous genes (PE26, PE27) were translocated between non-syntenic contig sequences of *A. lentis* and *A. rabiei* (Al-14 and Ar-9; Figure 4). Pairs of adjacently located homologous genes have likely originated by gene duplication. Whether these adjacently located paralogs have evolved different functions is unclear.

Homology between 17 SMCs of *A. lentis* and *A. rabiei* is shown in Figure 4, with links colored green. Fifteen regions identified as SMCs in *A. lentis* or *A. rabiei* shared sequence homology and were found in conserved syntenic regions between species and two SMCs appeared to be translocated when comparing synteny between species. Detailed analysis of the complement and arrangement of genes within clusters and their orthologs is outside the scope of this genome report. Nevertheless, the catalogue of genes likely responsible for the production of biologically active molecules in *A. lentis* and the differences between this and other *Ascochyta* species such as *A. rabiei*, will be important for the future studies of ascochyta diseases of crop legumes such as lentil and chickpea. The range of metabolites produced by pathogenic fungi such as those in the *Ascochyta* genus is dependent on the evolution and diversity of biosynthesis genes in different species and the taxonomy of species is correlated with the variation in secondary metabolite cluster genes, the proteins they encode and the metabolites synthesized (Kim *et al.* 2016, 2019).
